# Correcting systematic errors by hybrid 2D correlation loss functions in nonlinear inverse modelling

**DOI:** 10.1371/journal.pone.0284723

**Published:** 2023-04-20

**Authors:** Thomas G. Mayerhöfer, Isao Noda, Susanne Pahlow, Rainer Heintzmann, Jürgen Popp

**Affiliations:** 1 Leibniz Institute of Photonic Technology (IPHT), Jena, Germany; 2 Institute of Physical Chemistry and Abbe Center of Photonics, Friedrich Schiller University, Jena, Germany; 3 University of Delaware, Newark, DE, United States of America; National Institute of Technology, India (Institute of National Importance), INDIA

## Abstract

Recently a new family of loss functions called *smart error sums* has been suggested. These loss functions account for correlations within experimental data and force modeled data to obey these correlations. As a result, multiplicative systematic errors of experimental data can be revealed and corrected. The *smart error sums* are based on 2D correlation analysis which is a comparably recent methodology for analyzing spectroscopic data that has found broad application. In this contribution we mathematically generalize and break down this methodology and the *smart error sums* to uncover the mathematic roots and simplify it to craft a general tool beyond spectroscopic modelling. This reduction also allows a simplified discussion about limits and prospects of this new method including one of its potential future uses as a sophisticated loss function in deep learning. To support its deployment, the work includes computer code to allow reproduction of the basic results.

## Introduction

Systematic errors lead to non-accurate results with biases even if an experiment is repeated multiple times and the results are averaged to reduce the statistical random error. Systematic errors are often hard if not sometimes impossible to detect [1, 2]. One reason is that the down- or upshift of the mean compared to the true value does not influence the distribution of results caused by random errors. Even though it is possible to remove or reduce random errors and obtain a seemingly consistent (“precise”) result, these may nevertheless still be far from the underlying ground-truth.

In particular, in curve fitting, where experimental data points are fitted assuming that the points follow a mathematical or physical model, a good agreement between measured points and fitted curve may delude the operator into thinking that systematic errors are absent [2, 3]. A quantitative and objective measure of the agreement between experimental and fitted data is the residual sum of squares (RSS). This measure belongs to the so-called loss functions and is also used in machine learning procedures including deep learning to quantify the learning progress and, after finalization of the training phase, the quality of the model with help of a test set. In general, it must be kept in mind that a good agreement of a fit indicated by a low RSS is no sign of accuracy, neither in terms of the experimental data nor of the underlying model. That is, data analysis of quantitative experiments is based upon the assumption that the measured or calculated independent and dependent variables are not subject to systematic errors [4].

Curve fitting is often applied when nonlinear mathematical or physical inverse problems are to be solved that cannot be linearized. The inverse problem has its counterpart in the direct problem, which is a forward calculation, e.g., like the shadow cast by a 3D object on a white wall. The inverse problem, in contrast, involves reconstructing the 3D object from the shadow it casts, i.e., to derive the cause from the observation. Famous nonlinear inverse problems are the determination of properties of an object, based on the scattering characteristics of incoming radiation [5], recovering the diffusion coefficient of single phase fluid flows in porous media [6], or the recovering of wave-speeds and density distributions based on seismograms [7], but there are numerous further examples.

When linear inverse problems and those that can be linearized are to be solved, i.e., if the observable depends in a linear fashion on the independent variable(s), analytical solutions can be derived. Such linear models form the basis for many chemometric methods [8]. In contrast to linear problems, nonlinear problems require to iteratively improve the fitted curve by minimizing a measure of disagreement, to approach the curves of the experimental data. Potential applications of curve fitting are countless and encompass virtually all scientific disciplines. Examples include biosynthesis [9], thermoluminescence [10], solar energy [11], materials science and technology [12], agriculture [13], cancer research [14], kinetics [15], thermal engineering [16], transportation [17], soil science [18], remote sensing of ecosystems [19], epidemiology [20], power and energy engineering [21], population growth [22] and spectroscopy [23], to name just a few. The disagreement metrics to minimize during the fit depends on the properties of the noise and possibly on prior information on the parameters to fit. However, in most cases it is sensible to assume Gaussian statistics, which requires the minimization of the RSS also called the sum of squared errors or its weighted version in case of non-uniform but known variances. The RSS traces back to Carl Friedrich Gauß. For the most part it is used in its original form, and not much work has been done in general lately to improve it, except for a few particular applications. E.g., recently Korkmaz proposed a generalized RSS for multiple linear regression [24]. In addition, Zhang et al. suggested an integrated residual sum of squares for a supervised principal component regression method for relating functional responses with high dimensional predictors [25]. To fit high-dimensional data sets with more predictors than the sample size, Roozbeh et al. proposed penalization terms [26]. While these works were motivated by problems completely different to ours, Sumon and Majumder applied weighted versions of the RSS to account for systematic deviations, but in form of single data like irrelevant data, missing observation, and distributional faults [27].

Recently, we derived alternative loss functions, which are based on 2D correlation analysis or spectroscopy (2D COS) [3, 28]. 2D correlation analysis has been introduced in the 1980s by one of us as a tool for infrared spectroscopy which found widespread use also in other spectroscopic methods, like Raman spectroscopy and mass spectrometry [29]. In principle, 2D correlation spectroscopy is based on acquiring a series of spectra under a systematic change of one parameter of the sample (the so-called perturbation), e.g., the stretch of a polymer, the temperature or the concentration of one compound in a mixture. The perturbation can certainly also be a parameter of an established physical model to describe how its alteration induces changes in the spectra, like the thickness of films. A variant of 2D correlation spectroscopy is hybrid 2D correlation spectroscopy, which allows the comparison of two different spectral series, e.g., the same compound under two different perturbations. In the sense of curve fitting, a variant would be to let one series consist of experimental data, whereas the second comprises modelled spectra.

In the original 2D correlation maps, half of the data points are redundant, due to symmetry relations between points separated by the diagonal from low to high values of the independent variable. For hybrid 2D correlation maps these relations do no longer hold, but the more the two series resemble each other, the smaller the deviations from this symmetry relations become. This property of hybrid 2D correlation maps can be exploited by formulating an alternative criterion for the resemblance between experimental and modelled data which includes the correlations in between the series, which we call *smart error sum* (SES).

In contrast to the fits using the conventional RSS, the 2D correlation-based *smart error sum* does not force the modelled curve to agree point by point with experimental curves, but accounts for correlations between the latter and between the individual points of a curve. As a consequence, even when the experimental data are reduced by a (frequency dependent) factor, the data can still be analyzed in a meaningful way. In spectroscopy, such a situation is often encountered, for example, due to diffuse reflection caused by surface roughness, or measured data becomes larger than predictable by models which do not account for such experimental problems. This approach can not only detect, but also remove multiplicative systematic errors as has been demonstrated for infrared spectra of films on substrates with different thicknesses (additive systematic errors can also be treated after applying an exponential transformation).

Quite often, though, series of spectra are not available and it is only one data curve that is to be fitted. In this case inter spectral correlations cannot be exploited. However, it is still possible to use correlations between the individual data points based on a recent subtype of 2D correlation analysis. The so-called two-trace 2D correlation analysis or spectroscopy (2T2D COS) [30, 31] requires only two sets of spectral data of which only one needs to be experimental. In this case the symmetry relations cannot be used as a criterion, but if experiment and model agreed perfectly, one of the maps would amount to become everywhere zero. The value of this idea has been proven employing the same physical system as the original *smart error sum*. As we will show, the 2T2D SES approach is similar to utilizing normalized cross-correlation (NCC) and zero mean normalized cross-correlation (ZNCC) as SES. NCC and ZNCC are related to 2D correlation analysis [29] and often used for signal analysis. Examples entail comparing image quality in competition with the conventional residual sum of squares [32–35], tracking wavelength-shifts in Fiber-Bragg gratings [36, 37]. and, recently, also least squares optimizations of images [38].

While the application to real systems and problems helped to establish the validity of the approach, it also partially obscured the mathematical basis and the principal properties of the method. To enable broader application, we therefore here reduce it to its essential properties and demonstrate it based on a simple example in the following. In addition, we provide the 2T2D-based smart error sum in a form that scales, like the conventional sum of squared residuals, linearly with the number of points. Accordingly, the former can replace the latter in nonlinear curve-fitting applications that are prone to unknown systematic experimental errors. The code of the program which we used to obtain the results shown in the following is made available together with this work so that they can be easily reproduced. In addition, the code can effortlessly be modified to be used for other non-linear models.

## Contributions

The fundamental idea of this paper is to present the pure mathematical background of the methods from [3] and [28] in a form understandable to most readers independent of specialty and to investigate under which prerequisites the new family of loss functions can be applied. With regard to members of the family of smart error sums, the paper will introduce in the theoretical considerations section two novel members, namely the normalized cross correlation and the Pearson coefficient, which can also be used as loss functions after small, but important alterations, which are derived from an altered 2T2D loss function. The latter alteration, introduced in this paper, scales linearly with the number of points (very much like the conventional residual sum of squares) and no longer with the squared number of points as the previous. This immensely increases its potential, in particular for complex problems.

Concerning the requirements for the application of the new family of loss functions, we will show in the following that nonlinearity with respect to the external variable (“the perturbation”) is not sufficient to allow the *smart error sums* to be used. Instead, the correct criterium is a disproportionate change with the perturbation. This is a vital insight, because it proves that the *smart error sums* reject automatically approximations that do not lead to disproportionate changes. Finally, we will show how the *smart error sums* automatically reject unrealistic models. This could be of great interest for neuronal networks as it should allow to build models easier and thus faster.

## Theoretical considerations

### Basic equations for 2D-COS

The following is based on the matrix algebra employed for 2D-COS as used by Noda and Osaki [29]. Since the formalism was originally developed for spectroscopy, we have to slightly reformulate it. However, to allow the reader to connect the following to the original literature, we will try to adhere to the original terminology as closely as possible. We assume that we have a function ${\tilde{y}}_{k}={\tilde{y}}_{k}(x,t)$ of two variables *x* and *t* of which we call the former the shaping variable and the latter the perturbation. A number of different data points located on *m* different curves which differ with regard to *t*, shall be represented by employing discrete values *x*_*i*_ and *t*_*j*_ according to ${\tilde{y}}_{k}={\tilde{y}}_{k}(x_{i},t_{j})$. These curves will be called a set of dynamic spectra ${\tilde{y}}_{k}={\tilde{y}}_{k}(x_{i},t_{j})$ The dynamic spectra are arranged in a matrix **Y**_*k*_ in the following way:

$$Y_{k}=[\begin{array}{cccc} {\tilde{y}}_{k}(x_{1},t_{1}) & {\tilde{y}}_{k}(x_{2},t_{1}) & \ldots & {\tilde{y}}_{k}(x_{n},t_{1})\\ {\tilde{y}}_{k}(x_{1},t_{2}) & {\tilde{y}}_{k}(x_{2},t_{2}) & \ldots & {\tilde{y}}_{k}(x_{n},t_{2})\\ \ldots & \ldots & \ldots & \ldots \\ {\tilde{y}}_{k}(x_{1},t_{m}) & {\tilde{y}}_{k}(x_{2},t_{m}) & \ldots & {\tilde{y}}_{k}(x_{n},t_{m}) \end{array}].$$ (1)

The index *k*∈{1,2} and indicates if the set of dynamic spectra consists of either the set of “measured” (*k* = 1) or the set of simulated spectra (*k* = 2). One may think that it is advantageous to mean-center the dynamic spectra, i.e., subtracting the mean spectrum of the series from each individual measured spectrum. However, such mean-centering or, more general, referencing is often not only unnecessary [39], but sometimes even detrimental. Yet, if the array of curves or dynamic spectra share a common offset, this offset needs to be removed prior to application, otherwise not only the 2D correlation maps [40], but also the smart error sums are ill-defined.

From the matrices **Y**_*k*_ the variance-covariance matrices **Φ**_*xx*_ can be generated by:

$$\Phi_{xx}=\frac{1}{m-1}Y_{1}{}^{T}Y_{2}.$$ (2)

If **Y**_1_ = **Y**_2_, then we speak of conventional 2D-COS, whereas the case **Y**_1_ ≠ **Y**_2_ leads to a so-called hybrid correlation analysis. In case of the conventional smart error sum **Y**_1_ is formed from the “measured” and **Y**_2_ from the corresponding simulated curves. As pointed out in ref. [29], each element of the variance-covariance matrix expresses the similarity between a specific pair of intensity variations at different *x*_*j*_. If **Y**_1_ = **Y**_2_, the diagonal elements are the autocorrelation functions of the intensity variations along *t* at a given *x*_*j*_.

The variance-covariance matrix is identical to the synchronous 2D correlation map/spectrum. In order to compute the asynchronous 2D correlation map/spectrum, the Hilbert–Noda transformation matrix **N** must be calculated first. The elements of **N** are given by:

$$N_{ij}=\{\begin{array}{c} 0\;\mathrm{if}\;i=j\\ \frac{1}{\pi (j-i)}\;\mathrm{otherwise} \end{array}.$$ (3)

The elements of the asynchronous 2D correlation map/spectrum can then be calculated according to,

$$\Psi_{xx}=\frac{1}{m-1}Y_{1}{}^{T}NY_{2},$$ (4)

where we again distinguish between the conventional case (I) and hybrid 2D-COS (II).

### Derivation of the smart error sum

As already mentioned, in the introduction for the conventional (**Y**_1_ = **Y**_2_) 2D-correlation analysis for synchronous and asynchronous spectra/maps certain symmetry relationships hold. Accordingly, the synchronous spectra are always symmetric relative to the diagonal elements Φ(*x*_*i*_, *x*_*j*_). This condition can be expressed as,

$$\Phi (x_{j},x_{k})=\Phi (x_{k},x_{j}).$$ (5)

Accordingly, the diagonal from small to large *x* values is a mirror plane that relates each point above the diagonal to its mirror image below it. From Eq (5) it follows that the sum of differences of all variances and covariances above the diagonal and their counterparts below the diagonal are zero:

$$\sum_{k=1}^{l}\sum_{j=k}^{l}\left[\Phi (x_{j},x_{k})-\Phi (x_{k},x_{j})\right]=0.$$ (6)

For hybrid 2D-COS, the synchronous maps are not necessarily obeying the above condition. The residuals of the differences of the elements Φ(*x*_*j*_, *x*_*k*_) and Φ(*x*_*k*_, *x*_*j*_) is a measure of dissimilarity, which can be generally written as,

$$D_{S}^{p}=\sum_{k=1}^{l}\sum_{j=k}^{l}{\left[\Phi (x_{j},x_{k})-\Phi (x_{k},x_{j})\right]}^{p},$$ (7)

with *D*_*S*_, the so-called Minkowski distance, which is called the Euclidian distance for *p* = 2. Therefore, hybrid 2D correlation maps allow a derivation of these quantities simply from their symmetry relations [3].

For asynchronous 2D correlation maps, a similar relationship can be derived. Accordingly, similarly to Eq (5), we find from the condition that the conventional asynchronous 2D correlation maps are always antisymmetric with respect to the diagonal the following relation: [3]

$$\begin{array}{l} \Psi (x_{j},x_{k})=-\Psi (x_{k},x_{j})\\ \to \sum_{k=1}^{l}\sum_{j=k}^{l}\left[\Psi (x_{j},x_{k})+\Psi (x_{k},x_{j})\right]=0 \end{array}.$$ (8)

This relation leads for the hybrid 2D-correlation asynchronous map to:

$$D_{A}^{p}=\sum_{k=1}^{l}\sum_{j=k}^{l}{\left[\Psi (x_{j},x_{k})+\Psi (x_{k},x_{j})\right]}^{p}.$$ (9)

Note that for both, Eqs (7) and (9), we can include the diagonal since the terms with *j* = *k* are zero.

For *p* = 2, $D_{S}^{2}$ and $D_{A}^{2}$ are special residual sums of squares, which we call the synchronous and the asynchronous residual sum of squares, SRSS and ARSS. SRSS and ARSS can be combined ad hoc to the smart error sum (SES) according to:

ln(SRSS)+ln(ARSS)=SES. (10)

While only a few lines of code need to be exchanged to implement SES, compared to the conventional RSS which scales with O(N) SES scales with $O(N^{2})$, which is an obvious drawback in particular for larger datasets with a high number of datapoints. In addition, in cases where only a single measured spectrum is available for curve fitting, the smart error sum cannot be used, simply because it is not possible to generate a 2D correlation map from a single spectrum. For this case, we have introduced an alternative smart error sum based on hybrid 2T2D-COS, with one measured curve, while the other is the simulated one. Synchronous and asynchronous 2D correlation spectrum/map are then calculated by [30, 31],

$$\begin{array}{l} \Phi (x_{j},x_{k})=\frac{1}{2}[s(x_{j})\cdot s(x_{k})+m(x_{j})\cdot m(x_{k})]\\ \Psi (x_{j},x_{k})=\frac{1}{2}[s(x_{j})\cdot m(x_{k})-s(x_{j})\cdot m(x_{k})] \end{array},$$ (11)

wherein *m*(*x*_*j*_) = **Y**_1_(*x*_*j*_,*t*_*l*_) is the measured and *m*(*s*_*j*_) = **Y**_2_(*x*_*j*_,*t*_*l*_) the simulated curve with an arbitrary *t*_*l*_. Based on Eq (11), the hybrid synchronous spectrum is always symmetric and the asynchronous spectrum is always antisymmetric relative to the diagonal. Therefore, the underlying principle of the *smart error sum*, introduced for series of curves, namely to increase the symmetry of the hybrid synchronous 2D correlation map and the antisymmetry of the hybrid asynchronous 2D correlation map by varying the fit parameters, cannot be employed. As an alternative we can use that the asynchronous 2T2D-Correlation map Ψ_*xx*_ vanishes if both the given and the modelled curve are linearly dependent. Put in concrete terms, in this case the given and the modelled curve can also, as in case of the conventional *smart error sum*, differ by a simple scalar multiplication factor. Accordingly, the 2T2D *smart error sum* is given by [28],

$$D_{A2T}^{p}=\sum_{k=1}^{l}\sum_{j=k}^{l}{\left[\Psi (x_{j},x_{k})\right]}^{p},$$ (12)

where we set *p* = 2. Therefore, a corresponding algorithm would minimize $D_{A2T}^{2}$ by finding optimized values for the fit parameters. In Eq (12), all points below the diagonal need not to be considered, which follows from the asynchronous map being perfectly antisymmetric:

$${\left[\Psi ({\tilde{\nu }}_{j},{\tilde{\nu }}_{k})\right]}^{p}={\left(-1\right)}^{p}{\left[\Psi ({\tilde{\nu }}_{k},{\tilde{\nu }}_{j})\right]}^{p}.$$ (13)

On the other hand, if set *p* = 2, then there is a possibility to significantly simplify Eq (12), if we let both sums run from 1 to *l*. In this case,

$$\begin{array}{l} D_{A2T}^{2}=\sum_{k=1}^{l}\sum_{j=1}^{l}{\left[\Psi ({\tilde{\nu }}_{j},{\tilde{\nu }}_{k})\right]}^{2}\\ \;=\frac{1}{4}\sum_{k=1}^{l}\sum_{j=1}^{l}\left[s({\tilde{\nu }}_{j})s({\tilde{\nu }}_{j})\cdot m({\tilde{\nu }}_{k})m({\tilde{\nu }}_{k})+s({\tilde{\nu }}_{k})s({\tilde{\nu }}_{k})\cdot m({\tilde{\nu }}_{j})m({\tilde{\nu }}_{j})-2\cdot s({\tilde{\nu }}_{j})\cdot m({\tilde{\nu }}_{j})s({\tilde{\nu }}_{k})\cdot m({\tilde{\nu }}_{k})\right]\\ \;=\frac{1}{4}\left(\sum_{j=1}^{l}s({\tilde{\nu }}_{j})s({\tilde{\nu }}_{j})\sum_{k=1}^{l}m({\tilde{\nu }}_{k})m({\tilde{\nu }}_{k})+\sum_{k=1}^{l}s({\tilde{\nu }}_{k})s({\tilde{\nu }}_{k})\sum_{j=1}^{l}m({\tilde{\nu }}_{j})m({\tilde{\nu }}_{j})-2\sum_{j=1}^{l}m({\tilde{\nu }}_{j})s({\tilde{\nu }}_{j})\sum_{k=1}^{l}m({\tilde{\nu }}_{k})s({\tilde{\nu }}_{k})\right)\\ \;=\frac{1}{2}\left(\sum_{j=1}^{l}s{\left({\tilde{\nu }}_{j}\right)}^{2}\sum_{k=1}^{l}m{\left({\tilde{\nu }}_{k}\right)}^{2}-2{\left(\sum_{j=1}^{l}m({\tilde{\nu }}_{j})s({\tilde{\nu }}_{j})\right)}^{2}\right) \end{array}$$ (14)

which scales with O(N) like the conventional residual sum of squares instead of $O(N^{2})$ like the other smart error sums based on 2D correlation analysis while preserving the advantage that only a few lines of code has to be exchanged. The advantage of the smart error sums in comparison with the conventional residual sum of squares as minimalization criterion is that for the former experimental and simulated curve are not forced to agree by all means but can be different by an individual factor, the optimum of which is determined by maximum correlation. In other words, not only the best agreement between original and simulated values determines the fit parameters, but also the correlation of the curves in a series or within a curve. From a mathematical point of view, the additional degree of freedom can be understood in terms of the phase angle:

$$\Theta \left(x_{1},x_{2}\right)=\arctan\{\frac{\Psi (x_{1},x_{2})}{\Phi (x_{1},x_{2})}\}.$$ (15)

The term phase angle is used, because **Φ**_*xx*_ and **Ψ**_*xx*_ are linearly independent and can be described formally as a complex function:

$$X_{xx}=\Phi_{xx}+i\Psi_{xx}.$$ (16)

Θ(*x*_1_,*x*_2_) is then derived from the polar form. For hybrid 2T2D correlation analysis, Ψ(*x*_1_,*x*_2_) becomes zero if the original curve and its best fit agree within a multiplication factor, which means that Θ(*x*_1_,*x*_2_) = 0. This situation means that the two curves are linearly dependent or even identical if systematic errors are absent. Accordingly, an alternative form for the 2T2D-based *smart error sum* is given by: [28]

$$D_{A2T}^{p}=\sum_{k=1}^{l}\sum_{j=k}^{l}{\left[\Theta (x_{k},x_{j})\right]}^{p}.$$ (17)

For series-based hybrid 2D correlation analysis, the ratios Ψ_1_(*x*_1_,*x*_2_)/Φ_1_(*x*_1_,*x*_2_) for the set of the given curves and Ψ_2_(*x*_1_,*x*_2_)/Φ_2_(*x*_1_,*x*_2_) for the fitted curves are equal if the correlations within both sets of curves agree. An alternative form of the original smart error sum is therefore [28],

$$D_{SES}^{p}=\sum_{k=1}^{l}\sum_{j=k}^{l}{\left[\Theta_{ex}(x_{k},x_{j})-\Theta_{sim}(x_{k},x_{j})\right]}^{p}.$$ (18)

where Θ_*ex*_(*x*_*k*_,*x*_*j*_) are the phase angles of the original data and Θ_*sim*_(*x*_*k*_,*x*_*j*_) are those of the simulated curves. This form, for *p* = 2 and without consideration of its symmetry properties, has originally been introduced by Shinzawa et al. [41, 42] and used exclusively for the method of alternating least squares (ALS). In this form, a theoretical problem of Eq (10) is avoided, which occurs if either SRSS or ARSS becomes zero, which in practice unlikely happens due to numerical errors related to the conversion of numbers to the binary system. To be on the safe side, a dummy regularizing positive constant ε can be incorporated into Eqs (6) and (7), e.g. ε = 10^−10^ (this value is small enough to have no effect on the actual computation and may be viewed as a predictable substitute for random bit noise). Similar consideration may apply to the calculation of the phase angle defined in Eq (15) and it may be advantageous to regularize the denominator, even though the chance for the intensity of synchronous spectrum becomes exactly zero might be slim (there are chances that this can happen near the zero-crossing area. The sign of the regularization constant has to be the same as the sign of the synchronous spectrum intensity. The primary reason for regularizing the ratio between asynchronous and synchronous intensities is to avoid the ambiguity of the zero-divided-by-zero situation where the dynamic spectrum remains zero). Note that for a typical arctangent function routine, the direction of a vector in the phase plain is confined to the first and fourth quadrants. In other words, the phase angle calculated by a computer is automatically assumed to take the value between–π/2 and +π /2. In a practical physically expected situation, a phase vector can point to the direction outside of this artificial confinement. Therefore, it is generally advantageous to assume that the phase angle should be confined between -π/4 and +3π/4, and to add π whenever the calculated value lies between—π/2 and—π/4. Unfortunately, the form of Eq (18) prevents it from simplifications so that it scales with $O(N^{2})$ like the original SES.

According to Eq (18), individual 2D maps can differ by multiplication factors, even though their phase angles are equal. In the absence of systematic errors, the factor becomes unity. It might not be obvious, but the normalized cross correlation *NCC* coefficient can be derived from the 2T2D SES:

$$\sqrt{-\frac{D_{A2T}^{2}}{\sum_{j=1}^{l}s{\left({\tilde{\nu }}_{j}\right)}^{2}\sum_{k=1}^{l}m{\left({\tilde{\nu }}_{k}\right)}^{2}}-\frac{1}{2}}=\frac{\sum_{j=1}^{l}m({\tilde{\nu }}_{j})s({\tilde{\nu }}_{j})}{\sqrt{\sum_{j=1}^{l}s{\left({\tilde{\nu }}_{j}\right)}^{2}\sum_{k=1}^{l}m{\left({\tilde{\nu }}_{k}\right)}^{2}}}=NCC.$$ (19)

**Fig 1:**
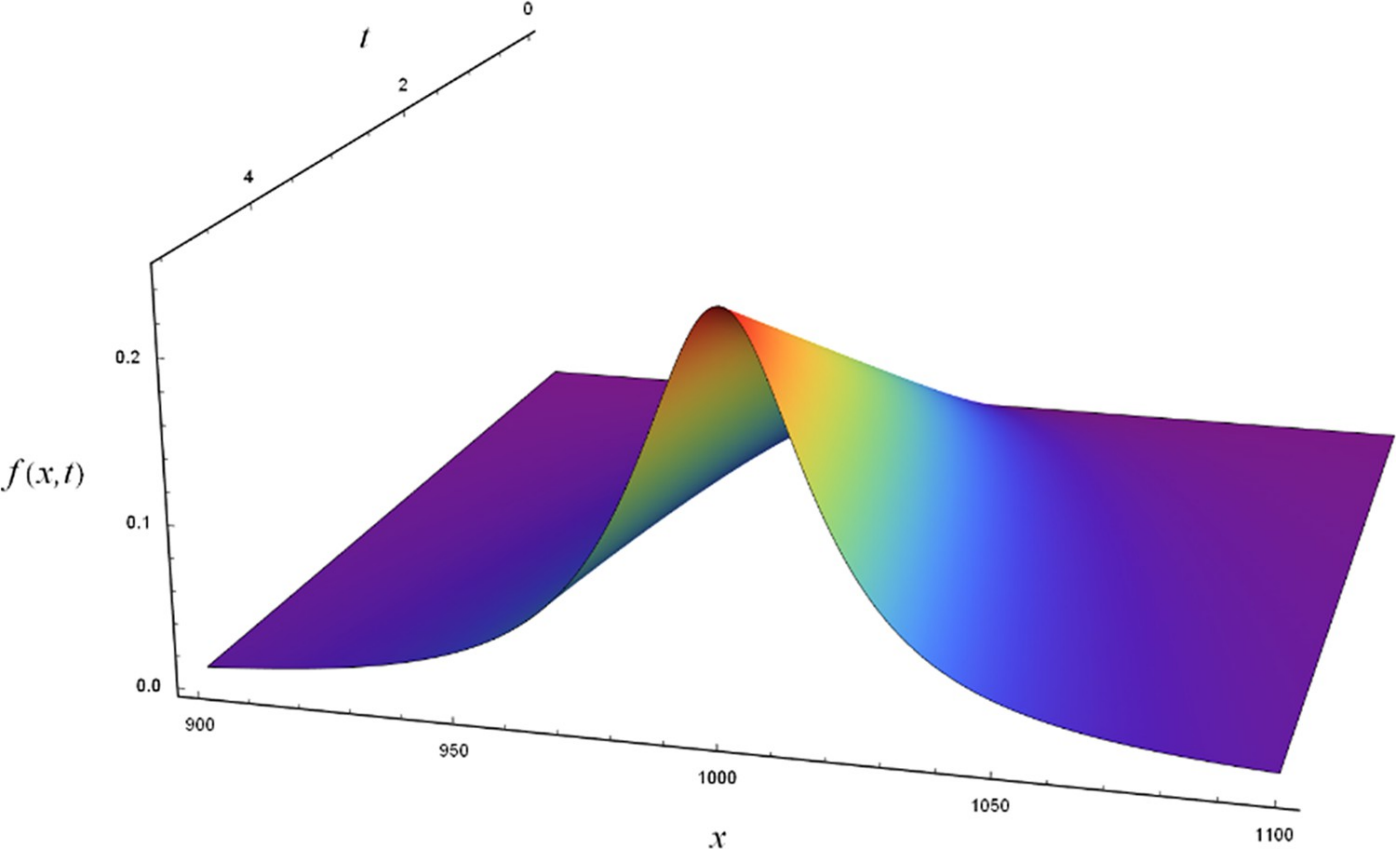
Visualization of Cauchy-type function used throughout this work. The curve depicted was generated with *x*_0_ = 1000, γ = 30, *a* = *b* = 0.

As long as *m* = *C*⋅*s*, with an arbitrary factor *C*, *NCC* = 1, otherwise −1<*NCC*<1. In other words, −*NCC* can also be employed as a loss function and then shares the property of the 2T2D *smart error sum* that the experimental and the simulated spectrum can differ by a factor. Quite often the *NCC* is used in a form that is mean-centered, to be more precise zero mean-centered, which is then called zero-mean normalized cross correlation *ZNCC*,

$$ZNCC=\frac{1}{l+1}\frac{\sum_{j=1}^{l}\left(m({\tilde{\nu }}_{j})-\mu_{m})(s({\tilde{\nu }}_{j})-\mu_{s}\right)}{\sigma_{m}\sigma_{s}},$$ (20)

where *μ*_*i*_ are the mean spectral intensities of *m* and *s* and *σ*_*i*_ are their standard deviations. Both, *NCC* as well as *ZNCC* share the advantage of the *2T2D SES* to scale with O(N) like the original RSS. In contrast to *NCC* and the 2T2D smart error sum, the *ZNCC* is additionally immune to offsets *O*: *m* = *C*⋅*s*+*O*. To show the main features of the smart error sums we use Cauchy-type model distributions of the general form,

$$f\left(x,t\right)=t\left(1+at\right)\frac{\gamma }{{\left(x-x_{0}-bt\right)}^{2}+\gamma^{2}},$$ (21)

to generate curves that we fit with the same type of functions. If the constants *a* and *b* are small, the function does not deviate noticeably from the Cauchy-distribution that is depicted in Fig 1. The larger *b* is, the more the maximum shifts to smaller *x*-values for increasing *t*. The parameter *a* induces a non-linear increase of the amplitude.

The fits were performed by a corresponding custom-made program based on Wolfram Mathematica® 10.3 which is provided as supporting information together with a variant programmed in Julia.

## Results and discussion

### Prerequisites for applying smart error sums

The crucial point for applying the smart error sum is the appearance or nature of the synchronous and asynchronous map or, alternatively, the map of the phase angles. For the smart error sum based on a series of curves for different perturbations *t* (Eq (10), methods), it is pivotal that the hybrid synchronous map is not already symmetrical to the diagonal from low to high *x*, as it is for non-hybrid maps, and that the hybrid asynchronous map is not zero. Unfortunately, this case is not uncommon, e.g., for the Cauchy-type functions if *b* equals zero (cf. Eq (21), methods). This may come as a surprise, because if you are familiar with 2D correlation spectroscopy, then you know that the asynchronous map is supposed to be nonzero if “the dynamic spectrum behaves nonlinearly with respect to the external variable”, i.e. the perturbation [29]. We can introduce such a nonlinearity by setting *a* ≠ 0 in Eq (21). But, as long as *b* = 0, the asynchronous map will remain zero everywhere, which does not change even if we multiply all curves with a constant factor to cause **Y**_1_ ≠ **Y**_2_. In fact, it seems that it is not a nonlinear change of *f*(*x*,*t*) in *t* that results in a non-zero asynchronous map, but the change must be disproportionate. Such a change can be induced by setting *b* ≠ 0, because then the maximum of the distribution downshifts increasingly if *t* increases.

**Fig 2:**
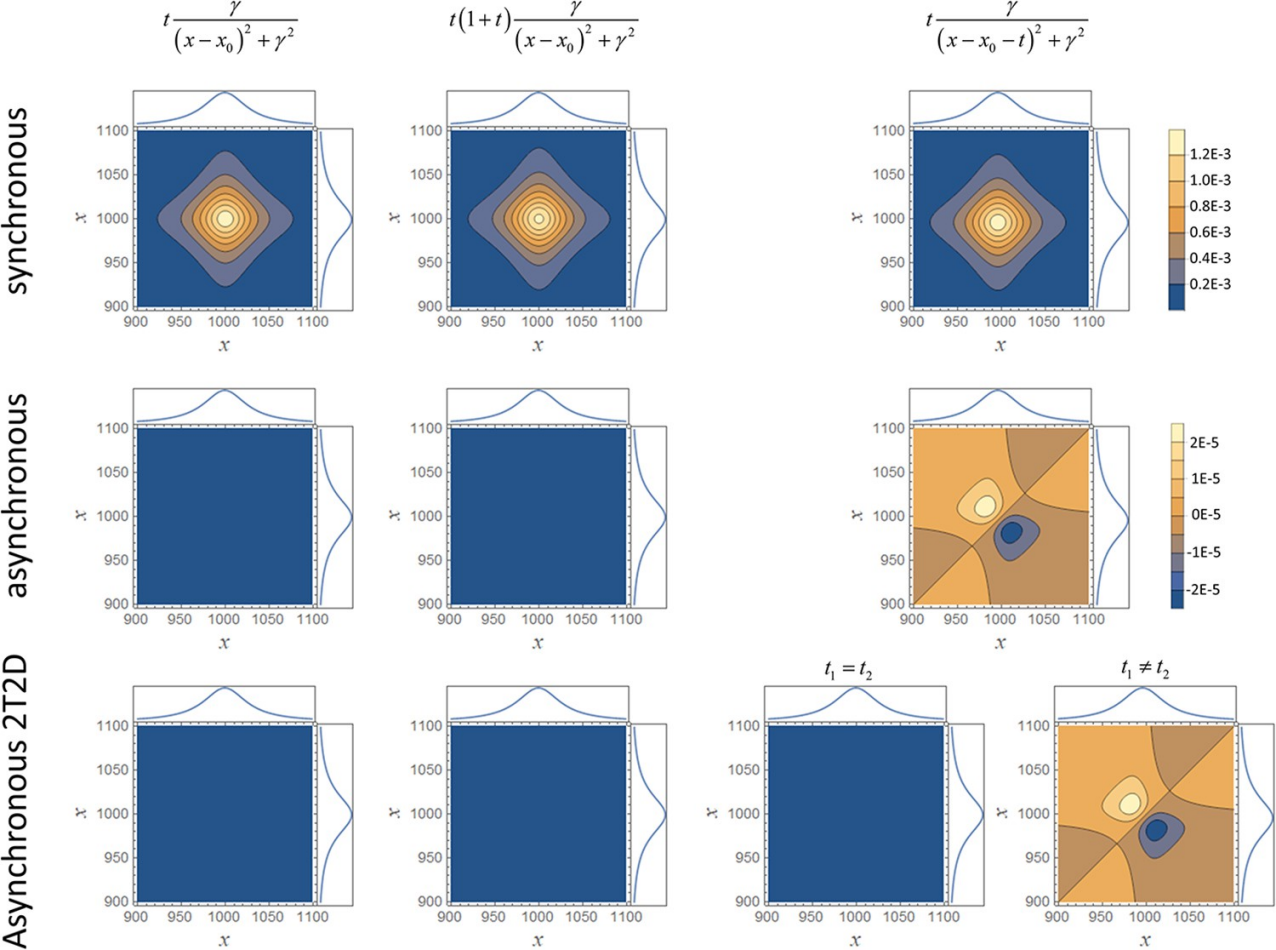
2D-Correlation maps of Cauchy-type functions. Comparison of the hybrid synchronous and asynchronous maps of the Cauchy-type functions used in this work (**Y**_1_ = **Y**_2_).

It looks like the presence of such a disproportionate change with increasing *t* is the criterion that must be fulfilled for the smart error sums to work, including the ones that are based on 2T2D-correlation as well as NCC (Eq (19)) and ZNCC (Eq (20)). Accordingly, the synchronous map presents the proportionate changes and the asynchronous map the disproportionate changes of *f*(*x*,*t*) with *t*. The different synchronous and asynchronous maps for the Cauchy-type functions are displayed in Fig 2. The synchronous maps are for all investigated functions quite similar. In contrast, the asynchronous maps stay zero for linear and quadratic (*a* = 1) proportionate changes, while the map becomes non-zero for disproportionate changes (*b* = 1) and shows a typical pair of cross-peaks indicating a shift of the peak maximum. The same holds true for the asynchronous 2T2D maps, except that two cases have to be distinguished for *b* = 1. These are the case *t*_1_ = *t*_2_, for which the asynchronous map is still zero, whereas it is similar to the conventional asynchronous map for the second case for which *t*_1_ ≠ *t*_2_. This agrees with the definition of the *smart error sum* in this case, since *t* itself can certainly be one of the fit parameters.

### Employing and comparing the smart error sums

In fact, the example that we want to showcase in the following is about fitting *t*. For a reader familiar with 2D correlation spectroscopy this may seem surprising, since *t* is usually assumed to simply increase systematically, which is important as otherwise semiquantitative deductions about the relative order of spectral changes are not possible. Note that 2D-COS can also be used in case of unevenly spaced increments of the perturbation, a corresponding extension of Eqs (2)–(4) for such increments has been provided [43]. If the sequence of the dynamic spectra is unknown, a computation of the asynchronous spectrum is not meaningful in contrast to the synchronous spectrum [44]. For applications of the smart error sum, however, neither equidistance nor the order of *t* values is of importance, which is why *t* can also be a fit parameter.

**Fig 3:**
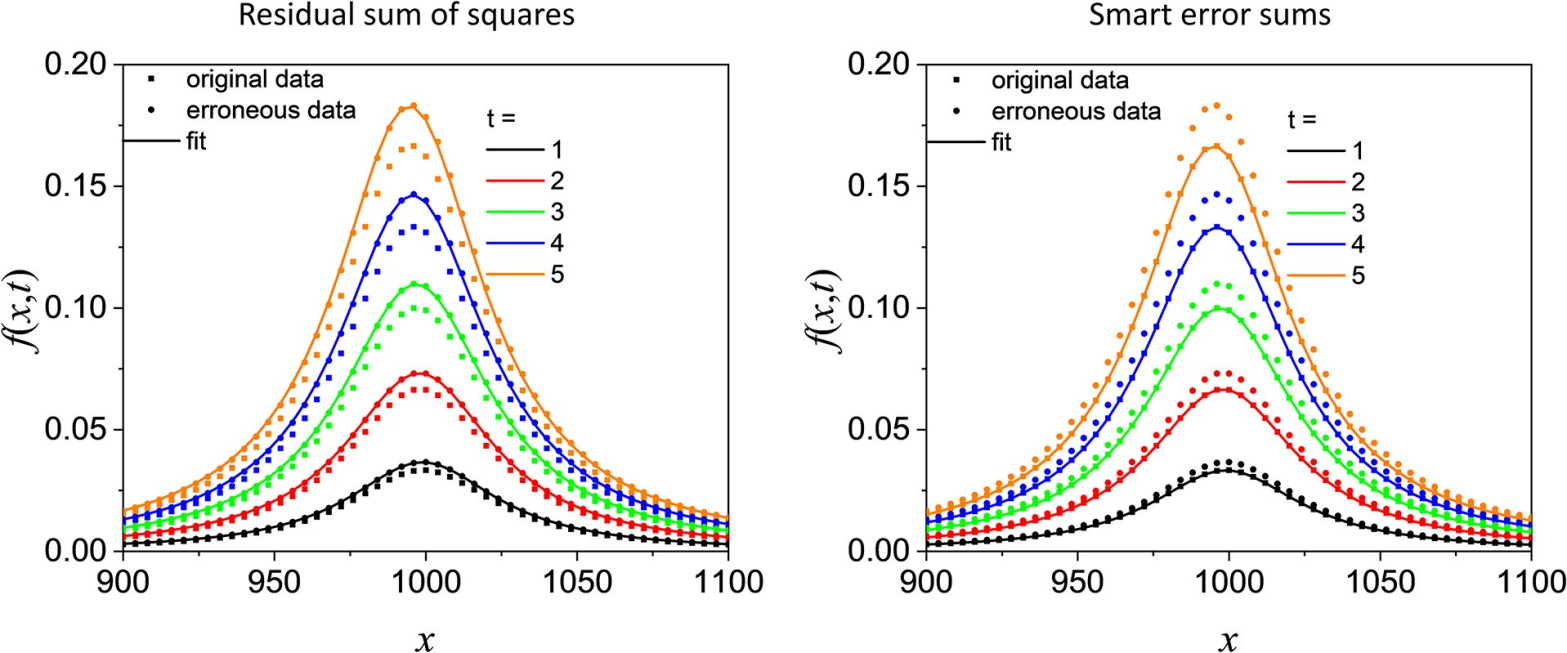
Comparison of performance for smart error sum and conventional error sum. Fit of the erroneous data with the conventional residual sum of squares (left panel) and with the smart error sums (right panel, the results obtained with the different error sums agree within line thickness except for Eq (18) if *t*∈{1,3}).

We nevertheless generated 5 curves by assuming *t*∈{1,2,3,4,5}, *x*_0_ = 1000 and γ = 30 in the range of 900<*x*<1100 and added 10% systematic error by multiplying the curves by 1.1. Note that this is an oversimplification of a real situation, where the factor would depend on *x*–otherwise it would simply be possible to correct the spectra by introducing this factor as an additional fit parameter, but here we focus on demonstrating the method in a simple setting. The erroneous data were subsequently fitted by employing the conventional sum of squares and with the different smart error sums. The results are depicted in Fig 3 and in Table 1. Obviously, apparently perfect fits are possible employing the conventional sum of squares by adjusting the parameter *t*.

**Table 1:** Comparison of the fit results for different error sums. The first column refers to the equation of the corresponding error sum used for the fit (cf. section *Derivation of the smart error sum*). The following columns present the fit results for the different perturbations t_j_ based on the different errors sums and the relative time needed to complete the fits.

Equation	t_1_	t_2_	t_3_	t_4_	t_5_	Relative timing
**(1)**	1.09993	2.19944	3.29812	4.39557	5.4914	1
**(10)**	1.00067	1.99862	3.00092	3.99983	4.99997	105.9
**(12)**	1	2	3	4	5	29.5
**(14)**	0.99983	2.00001	3.00001	4	4.99999	2.3
**(17)**	1	2	3	4	5	46.4
**(18)**	0.956611	2.02517	2.95784	4.00158	4.99879	110.2
**(19)**	0.999966	2.00002	3.00002	4	5	11.0
**(20)**	0.999999	1.99999	3	4	5	62.5

Note that for the 2T2D smart error sums following Eqs (12) and (17), for which the latter is based on the phase angle, the convergence is fast enough so that the original *t* values are virtually recovered. While Eq (14) allows a much faster fit, Mathematica returns a slightly worse result. The convergence is much slower for the series based smart error sums. In principle, instead of adding the logarithm of SRSS and ARSS, an alternative for connecting both residual sums would be to use the product (in case of an addition, a weighting would be necessary, since the ARSS usually is several orders of magnitude smaller). However, we found that the convergence would be much worse, which is why we prefer Eq (10) over using the product of SRSS and ARSS (the use of either alone further deteriorates the result, in particular if SRSS is used).

In particular the phase angle-based smart error sum, Eq (18), has a comparably slow convergence, so that the fitted values differ considerably from the original values with the default settings of Mathematica’s NMinimize, even though we added the condition that the solutions must be in the interval of ±1 of the original value (cf. Table 1). Obviously, although it points to the correct values, the phase angle and series based smart error sum has by far the worst convergence independent of which of Mathematica’s built-in methods is chosen (“Nelder-Mead” [45], "DifferentialEvolution" “SimulatedAnnealing” and “RandomSearch”). The conventional correlation-based smart error sums NCC (Eq (19)) and ZNCC (Eq (20)) are slower than the faster 2T2D smart error sum. Not only do they show a somewhat inferior convergence, in real life applications where the error does not consist of a multiplicative error that is independent of *x*, they show also an inferior performance due to normalization. In particular ZNCC does not show any advantage compared to an also zero mean-centered and normalized residual sum of squares (not shown), which is in line with its poorer performance compared to NCC for pattern recognition in image analysis [46].

For the conventional residual sum of squares, the *t* values completely reflect the error of the data (cf. Table 1), but the nearly perfect adaption to the altered data belies about the failure and can cheat the user into believing that the parameters obtained from the fit are errorless. Not only that, it can even mislead the user into believing that the model that is applied is correct. As mentioned above, if *b* ≠ 0, then the maximum of the curves is increasingly shifted to lower *x* with increasing *t*. Nevertheless, it is also possible to fit the curves under the assumption *b* = 0 if *x*_0_ is allowed to be one of the fit parameters–in this case the fit of the erroneous curve is perfect for *x*_0_ = {999,998,997,996,995} and *t* = {1.1,2.2,3.3,4.4,5.5}, although the employed fit function is wrong.

### Application limits

**Fig 4:**
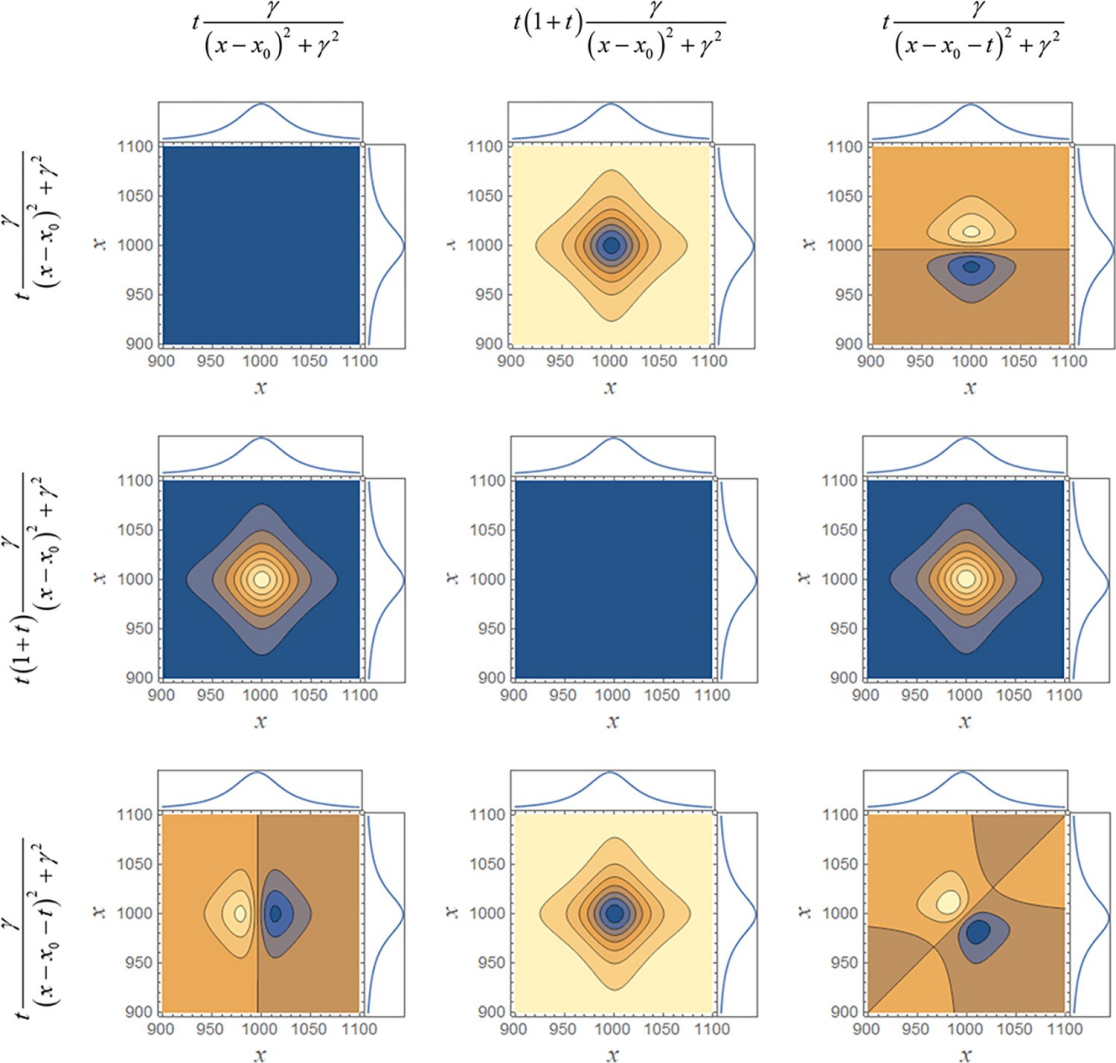
Asynchronous 2D correlation maps for the mixture of different functions. For all parameters (except the constants *a* and *b*) the same values were chosen for both series.

If smart error sums are employed, the erroneous situation mentioned at the end of the last section cannot occur, because in this case the fit cannot converge to a result. Hybrid 2D correlation analysis reveals the reason for this “failure” as can be seen in Fig 4. For mixtures of different functions, the asynchronous maps do not show the expected form, i.e., the values above the diagonal from small to large *x* do not have in general the inverted sign of the values below the diagonal, even though for both series the parameters, with the exception of the constants *a* and *b*, were the same. As a consequence, fits employing one of the smart error sums must fail, since the antisymmetry of the asynchronous map with respect to the diagonal can never be reached. In other words, if the use of a smart error sum for the fit of a physical problem leads to convergence, the used theoretical model must be adequate for the problem. On the other hand, if a fit fails because the asynchronous map does not show the expected distribution of signs, the applied model is not adequate for the problem at hand.

To present a concrete and practical example, Cauchy-functions are assumed in large parts of the spectroscopic community to describe the absorption of light, i.e., absorbance peaks. In fact, Lorentz derived based on dispersion theory that such profiles (therefore they are often called Lorentz-profiles) are good approximations for weak oscillators [47]. In the Lorentz-profile, however, the band position is decoupled from the peak intensity, in contrast to dispersion theory (the coupling results from the polarization of matter by light). If a single band is assumed it can be shown that *b* = 1/3 [48]. If the conventional residual sum of squares is used, the Lorentz-profile can nevertheless be employed to fit the bands–as long as no series is fitted, which is the usual case, a band shift is simply compensated by changing the peak position as in the example discussed above. In contrast, a fit employing one of the smart error sums cannot succeed. The simple reason is that the asynchronous map, and, with it, the map of the phase angles, is not antisymmetric in the sense that the values above the diagonal have in general the opposite value of those below the diagonal, if the model that was used to generate the original curves is different from the one the simulation is based on. Note that 2T2D maps behave differently and cannot be used to evaluate functional relations.

Again, it must be stated that this property can only be advantageously exploited for complex underlying laws that lead to disproportionate changes due to the perturbation. On the other hand, all less-complex relationships can be linearized and treated with the method of linear least squares, which provides analytical solutions. Therefore, curve fitting is not required.

## Conclusions

The family of correlation-based loss functions, called *smart error sums*, are important alternatives to the standard loss function for nonlinear fitting problems, the residual sum of squares. Strictly speaking, the latter should only be applied to data that do not contain systematic errors. If this condition is not obeyed, meaningful results cannot be expected. In contrast, using a *smart error sum*, does not lead to an equalization of experimental and modelled curve at all costs. Multiplicative factors, even those that are slightly depending on the independent variable, are acceptable and correlations like similar curve shapes are usually preserved. The disadvantage of 2D-correlation-based smart error sums is that they generally scale unfavorable with the square of the number of data points and the original version required a series of data, e.g., a series of spectra in optical modelling. In particular if the 2T2D *smart error sum* is employed, however, the fitting problem scales linearly with the number of data points, like the conventional residual sum of squares. In general, it is very simple to replace the latter by a correlation-based loss function in computer code, only a few lines have to be replaced. Linear fitting problems cannot be successfully approached, since all straight lines parallel to the solution have the same correlation. But such problems can be analytically solved and do not require advanced fitting algorithms with smart loss functions. More importantly, such problems also do not require to employ neural networks/deep learning, methods which are specifically suitable to solve nonlinear inverse problems, in particular those for which functional dependencies are (yet) unknown. For deep learning, on the other hand, it should be helpful to use loss functions based on smart error sums, because the latter obviously help to develop the correct functional relationships. In this stage this remains speculative, but we think that such loss functions do also support the training of neural networks as they can be effective measures to prevent underfitting and overfitting.

## Supporting information

S1 File Mathematica notebook for application of the smart error sum. This file can be used to reproduce all results presented in this paper by inserting the parameters for the variables given in the manuscript or adapted for applying the smart error sum to your own project.
(NB) Click here for additional data file.

S2 File Julia script for application of the smart error sum. This file can be used to reproduce all results presented in this paper by inserting the parameters for the variables given in the manuscript or adapted for applying the smart error sum to your own project. Julia is an open source project. More information can be found under https://julialang.org.
(JL) Click here for additional data file.
